# Supplementary material for: An open-source framework for large-scale, flexible evaluation of biomedical text mining systems
Source: J Biomed Discov Collab. 2008 Jan 29;3:1. doi: 10.1186/1747-5333-3-1 (PMC2276192; doi:10.1186/1747-5333-3-1)
Supplement: Additional file 1 — Evaluation platform source code. The additional file contains the software evaluation framework discussed in this paper. The most current release can be downloaded from SourceForge.net [1]. See the accompanying README file for instructions on installation and use. Also included in the distribution is a collection of UIMA wrappers for some commonly used BioNLP tools and annotated corpora. [file 1747-5333-3-1-S1.zip › bionlp_uima_repository-1.0.1/docs/api/edu/uchsc/ccp/util/nlp/mention/SlotMentionTypes.html]

SlotMentionTypes (Center for Computational Pharmacology Util/UIMA API)


|  |  |  |
| --- | --- | --- |
| **Overview** \| **Package** \| **Class** \| **Tree** \| **Deprecated** \| **Index** \| **Help** | |  |
| **PREV CLASS**   NEXT CLASS | **FRAMES**    **NO FRAMES**     **All Classes** |  |
| SUMMARY: NESTED \| FIELD \| CONSTR \| METHOD | DETAIL: FIELD \| CONSTR \| METHOD |  |


---


## edu.uchsc.ccp.util.nlp.mention Interface SlotMentionTypes

---

``` public interface SlotMentionTypes ```

---

| **Field Summary** | |
| --- | --- |
| `static String` | `BIOLOGICAL_SEQUENCE_SEQUENCE`             for Mutation project |
| `static String` | `CLAUSE_LEVEL` |
| `static String` | `CLAUSE_NUMBER` |
| `static String` | `CLAUSE_TYPE` |
| `static String` | `DOCUMENT_SECTION_NAME`             Document Section and SubSection slots |
| `static String` | `DOCUMENT_SUBSECTION_NAME` |
| `static String` | `EXPRESSION_EXPRESSED_GENE_OR_GENE_PRODUCT` |
| `static String` | `EXPRESSION_LOCATION` |
| `static String` | `FIGURE_ID` |
| `static String` | `MEDLEE_ID`             for MedLEE |
| `static String` | `MESH_ID`             for Olga's concept recognizers |
| `static String` | `NCBI_TAX_ID`             For Species Classification |
| `static String` | `PART_OF` |
| `static String` | `PHRASE_LEVEL` |
| `static String` | `PHRASE_NUMBER` |
| `static String` | `PHRASE_TYPE` |
| `static String` | `PROCESSED_TEXT_SLOT`             Document-level Slot Mentions |
| `static String` | `PROTEIN_ENTREZGENEID`             Semantic Slot Mentions |
| `static String` | `SLOT_MENTION_TYPE`             SlotMention Identifier - if this changes, Annotation DB must be adjusted accordingly |
| `static String` | `TABLE_ID` |
| `static String` | `TOKEN_IS_ANAPHOR_FOR` |
| `static String` | `TOKEN_LEMMA` |
| `static String` | `TOKEN_NUMBER` |
| `static String` | `TOKEN_PARTOFSPEECH`             Syntactic Slot Mentions |
| `static String` | `TOKEN_STEM` |
| `static String` | `TOKEN_TYPED_DEPENDENCY` |

**Field Detail**

### SLOT\_MENTION\_TYPE

```
static final String SLOT_MENTION_TYPE
```

:   SlotMention Identifier - if this changes, Annotation DB must be adjusted accordingly

    **See Also:**: Constant Field Values

---


### PROCESSED\_TEXT\_SLOT

```
static final String PROCESSED_TEXT_SLOT
```

:   Document-level Slot Mentions

    **See Also:**: Constant Field Values

---


### TOKEN\_PARTOFSPEECH

```
static final String TOKEN_PARTOFSPEECH
```

:   Syntactic Slot Mentions

    **See Also:**: Constant Field Values

---


### TOKEN\_LEMMA

```
static final String TOKEN_LEMMA
```

**See Also:**: Constant Field Values

---


### TOKEN\_STEM

```
static final String TOKEN_STEM
```

**See Also:**: Constant Field Values

---


### TOKEN\_NUMBER

```
static final String TOKEN_NUMBER
```

**See Also:**: Constant Field Values

---


### TOKEN\_IS\_ANAPHOR\_FOR

```
static final String TOKEN_IS_ANAPHOR_FOR
```

**See Also:**: Constant Field Values

---


### TOKEN\_TYPED\_DEPENDENCY

```
static final String TOKEN_TYPED_DEPENDENCY
```

**See Also:**: Constant Field Values

---


### PHRASE\_TYPE

```
static final String PHRASE_TYPE
```

**See Also:**: Constant Field Values

---


### PHRASE\_LEVEL

```
static final String PHRASE_LEVEL
```

**See Also:**: Constant Field Values

---


### PHRASE\_NUMBER

```
static final String PHRASE_NUMBER
```

**See Also:**: Constant Field Values

---


### CLAUSE\_TYPE

```
static final String CLAUSE_TYPE
```

**See Also:**: Constant Field Values

---


### CLAUSE\_LEVEL

```
static final String CLAUSE_LEVEL
```

**See Also:**: Constant Field Values

---


### CLAUSE\_NUMBER

```
static final String CLAUSE_NUMBER
```

**See Also:**: Constant Field Values

---


### PROTEIN\_ENTREZGENEID

```
static final String PROTEIN_ENTREZGENEID
```

:   Semantic Slot Mentions

    **See Also:**: Constant Field Values

---


### EXPRESSION\_LOCATION

```
static final String EXPRESSION_LOCATION
```

**See Also:**: Constant Field Values

---


### EXPRESSION\_EXPRESSED\_GENE\_OR\_GENE\_PRODUCT

```
static final String EXPRESSION_EXPRESSED_GENE_OR_GENE_PRODUCT
```

**See Also:**: Constant Field Values

---


### PART\_OF

```
static final String PART_OF
```

**See Also:**: Constant Field Values

---


### BIOLOGICAL\_SEQUENCE\_SEQUENCE

```
static final String BIOLOGICAL_SEQUENCE_SEQUENCE
```

:   for Mutation project

    **See Also:**: Constant Field Values

---


### DOCUMENT\_SECTION\_NAME

```
static final String DOCUMENT_SECTION_NAME
```

:   Document Section and SubSection slots

    **See Also:**: Constant Field Values

---


### DOCUMENT\_SUBSECTION\_NAME

```
static final String DOCUMENT_SUBSECTION_NAME
```

**See Also:**: Constant Field Values

---


### TABLE\_ID

```
static final String TABLE_ID
```

**See Also:**: Constant Field Values

---


### FIGURE\_ID

```
static final String FIGURE_ID
```

**See Also:**: Constant Field Values

---


### MESH\_ID

```
static final String MESH_ID
```

:   for Olga's concept recognizers

    **See Also:**: Constant Field Values

---


### MEDLEE\_ID

```
static final String MEDLEE_ID
```

:   for MedLEE

    **See Also:**: Constant Field Values

---


### NCBI\_TAX\_ID

```
static final String NCBI_TAX_ID
```

:   For Species Classification

    **See Also:**: Constant Field Values


---


|  |  |  |
| --- | --- | --- |
| **Overview** \| **Package** \| **Class** \| **Tree** \| **Deprecated** \| **Index** \| **Help** | |  |
| **PREV CLASS**   NEXT CLASS | **FRAMES**    **NO FRAMES**     **All Classes** |  |
| SUMMARY: NESTED \| FIELD \| CONSTR \| METHOD | DETAIL: FIELD \| CONSTR \| METHOD |  |


---
